# Nov/Ccn3, a Novel Transcriptional Target of FoxO1, Impairs Pancreatic β-Cell Function

**DOI:** 10.1371/journal.pone.0064957

**Published:** 2013-05-21

**Authors:** Renée Paradis, Noureddine Lazar, Peter Antinozzi, Bernard Perbal, Jean Buteau

**Affiliations:** 1 Department of Medicine, Université Laval, Quebec, Canada; 2 Unité de formation et de recherche en Biochimie, Université de Paris 7-D Diderot, Paris, France; 3 Department of Biochemistry, Wake Forest University School of Medicine, Winston-Salem, North Carolina, United States of America; University of British Columbia, Canada

## Abstract

Type 2 diabetes is characterized by both insulin resistance and progressive deterioration of β-cell function. The forkhead transcription factor FoxO1 is a prominent mediator of insulin signaling in β-cells. We reasoned that identification of FoxO1 target genes in β-cells could reveal mechanisms linking β-cell dysfunction to insulin resistance. In this study, we report the characterization of *Nov/Ccn3* as a novel transcriptional target of FoxO1 in pancreatic β-cells. FoxO1 binds to an evolutionarily conserved response element in the *Ccn3* promoter to regulate its expression. Accordingly, CCN3 levels are elevated in pancreatic islets of mice with overexpression of a constitutively active form of FoxO1 or insulin resistance. Our functional studies reveal that CCN3 impairs β-cell proliferation concomitantly with a reduction in cAMP levels. Moreover, CCN3 decreases glucose oxidation, which translates into inhibition of glucose-stimulated Ca^2+^ entry and insulin secretion. Our results identify CCN3, a novel transcriptional target of FoxO1 in pancreatic β-cells, as a potential target for therapeutic intervention in the treatment of diabetes.

## Introduction

The increasing prevalence of type 2 diabetes is cause for concern, and has spurred efforts to identify novel peptides with valuable properties for diabetes treatment [1]. Type 2 diabetes is characterized by both resistance of target tissues to the actions of insulin and impaired β-cell function [2], [3]. Studies in genetically modified mice have suggested that defects in insulin/IGF signaling in the β-cell contribute to β-cell failure [4], thereby establishing a causal link between insulin resistance and impaired β-cell function. One attractive scenario is that insulin and IGFs exert their effects through a common effector, acting on DNA transcription in β-cells [5].

Forkhead box (Fox)-containing transcription factors of the O sub-class (FoxO) are prominent transcriptional effectors of insulin and IGF signaling in β-cells [6]. FoxO1 inhibits β-cell proliferation in insulin-resistant states [7] as well as in response to growth factors [8], protects β-cells against hyperglycemia-induced oxidative stress [9], and controls energy metabolism in β-cells [10]. In view of the role of FoxO1 in β-cell compensation to insulin resistance [11], we reasoned that investigation of FoxO1 target genes could reveal mechanisms underlying β-cell failure in the context of insulin resistance. To this end, we carried out gene profiling analyses in INS832/13 cells [10]. Our genomic analysis led to the identification of *nephroblastoma overexpressed gene* (*Nov,* also known as *Ccn3*) as a novel FoxO1 target. The role of *Ccn3* in β-cells has never been explored.

The *Ccn3* gene was first identified in avian nephroblastomas as an integration site of the avian myeloblastosis-associated virus 1-N [12]. It encodes a peptide hormone that belongs to the CCN (Cyr61, CTGF, Nov) family [13], [14]. These hormones share a common structural homology [12]. As other members of the CCN family, CCN3 plays a role in various cellular processes including proliferation, adhesion, and differentiation [15]. The fact that the *Ccn3* gene is strongly up-regulated in response to FoxO1 activation and that it maps to a susceptibility locus controlling β-cell function in linkage studies of diabetic patients [16]–[20] prompted us to examine its role in the β-cell. Indeed, the biological role of CCN3 proteins in β-cells has never been explored. In the present study, we conducted a comprehensive biochemical analysis of *Ccn3* expression and action. Our results indicate that *Ccn3* is a transcriptional target of FoxO1. Its expression is increased in mice with FoxO1 gain-of-function as well as in insulin resistant mice. We also show that *Ccn3* expression in the pancreas is restricted to ducts and islet cells. Finally, CCN3 decreases both β-cell replication and insulin secretion.

## Materials and Methods

### Ethics statement

Animal work: This study was carried out in strict accordance with the recommendations of the Canadian Council on Animal Care. The protocol was approved by the Ethics Committee of Laval University.

### Reagents

RPMI 1640 and cell culture supplements, including fetal calf serum (FCS), were purchased from Life Technologies (Burlington, ON). Anti-CCN3 antibodies targeting the C-terminal region of the protein (K19M) were described previously [21]. Full-length human CCN3 protein were purchased from R&D systems (Minneapolis, MN).

### Cell Culture

INS832/13 cells [22] (passages 46–70) were grown in monolayer cultures in regular RPMI 1640 medium supplemented with 10 mmol/l HEPES, 10% FCS, 2 mmol/l L-glutamine, 1 mmol/l sodium pyruvate, 50 µmol/l β-mercaptoethanol at 37°C in a humidified (5% CO_2_, 95% air) atmosphere.

### Recombinant adenovirus infection

The constitutively active (CA) FoxO1 mutant carries single amino acid substitutions replacing the three main phosphorylation sites, Thr^24^-Ala, Ser^253^-Asp and Ser^316^-Ala and has been described previously [6]. Cells were seeded 2 days before use in 100 mm Petri dishes and cultured as described above. Cells were then infected with Ad-CAFoxO1 or Ad-β-galactosidase (Ad-βGal) at a MOI of 50 pfu/cell for 1 hr in 1 ml of complete medium. The viral solution was then replaced with complete medium and cells were allowed to recover for 24 hr before the experiment.

### Gene expression

RNA was isolated using the RNeasy mRNA kit (Qiagen, Mississauga, ON) and cDNA were synthesized using a reverse transcription kit from Life Technologies (Burlington, ON) following manufacturer's instructions. mRNA transcript levels were measured using a Rotor Gene 3000 system (Montreal Biotech, Montreal, QC, Canada). Chemical detection of the PCR products was achieved with SYBR Green I (Life Technologies, Burlington, ON). At the end of each run, melt curve analyses were performed, and a few samples representative of each experimental group were run on agarose gel to ensure the specificity of the amplification. Results are expressed as the ratio between the expression of the target gene and actin. In addition, we have used a RT2 profiler PCR array (Qiagen, Mississauga, ON) to analyze expression of a focused panel of genes controlling cell cycle progression, following the manufacturer’s protocol.

### Promoter assay

To investigate the effect of FoxO1 on *Ccn3* promoter activity, cells were co-transfected with a plasmid encoding either CAFoxO1 or control βGal, concomitantly with a Chloramphenicol acetyltransferase (CAT) reporter construct driven by the human Ccn3 promoter (∼900 bp upstream of the TSS, described in [23]). Lipofectamine 2000 (Life Technologies, Burlington, ON) was used as the transfection reagent and the plasmids were premixed in a 1∶3 ratio (CAT:CAFoxO1 or βGal) to ensure that all CAT-transfected cells would also express CAFoxO1 or βGal. The next day, cell lysates were prepared for CAT assays using a commercial ELISA kit (Roche, Laval, QC).

### Chromatin immunoprecipitation

Chromatin immunoprecipitation assay was performed with a commercially available kit from Millipore (Billerica, MA) according to manufacturer’s protocol. In brief, 1×10^6^ INS823/13 cells were either transduced with Ad-βGal or Ad-CAFoxO1 in complete RPMI medium, fixed in 1% formaldehyde, washed and resuspended in lysis buffer. Alternatively, FoxO1 was activated via serum deprivation overnight. Samples were sonicated to shear DNA to lengths between 200 and 500 bp. FoxO1/DNA complexes were immunoprecipitated with an anti-FoxO1 antibody (Santa Cruz, Santa Cruz, CA) and washed. DNA was recovered and amplified by PCR using oligonucleotides flanking the indicated promoter regions.

### Western blot

Cells were grown and incubated as described above, washed twice with PBS and lysed in 1 ml of ice-cold lysis buffer (50 mM Tris-HCl (pH 8.0), 1% Triton X-100, 150 mM NaCl, 1 mM PMSF, 1 µg/ml aprotinin, 5 mM sodium pyrophosphate, and 1 mM orthovanadate) for 30 min at 4°C. Conditioned media were collected following culturing INS832-13 cells in serum-free media for 24h. Precipitation of protein from conditioned culture medium was performed by methanol precipitation. Protein concentrations were determined using the Pierce BCA protein assay (Rockford, IL) and samples were resolved on 8% or 10% polyacrylamide gels.

### Immunohistochemistry

Cells were seeded in 6-well plates at 80% confluency, attached onto polyornithine-coated coverslips, and cultured as described above. Cells were then washed, fixed in paraformaldehyde and incubated with a cocktail of primary antibodies comprising rabbit anti-CCN3, mouse anti-Synaptobrevin (Princeton, NJ) and guinea-pig anti-insulin antibodies (Dako, Carpinteria, CA) working solution. Pancreas samples were fixed in paraformaldehyde and embedded in paraffin. We mounted 10 µm sections on slides and performed immunohistochemistry with anti-CCN3 antibodies.

### siRNA-mediated knockdown of Ccn3

Pre-validated Ccn3 siRNAs and control (scrambled) siRNAs were purchased from Ambion (Austin, TX) and were transfected using Lipofectamine RNAiMAX Life Technologies (Burlington, ON) following manufacturer’s protocol.

### Cell proliferation

INS832/13 cell proliferation was evaluated using BrdU cell proliferation ELISA kit from Roche (Laval, QC) according to manufacturer’s protocol. In brief, INS832/13 cells were seeded in 96-well plates (8×10^4^ cells/well) and allowed to recover for 24 hr. Subsequently, cells were incubated at low (5 mM) or high (25 mM) glucose in the absence or presence of serum for 24 h. BrdU was added to the culture medium for 1 hr. Cells were then fixed, incubated with a peroxydase-conjugated anti-BrdU antibody and the immune complexes were quantified by measuring the absorbance at the respective wavelength using a scanning multi-well spectophotometer (Biorad, Hercules, CA).

### Cyclic AMP determination

Measurements of intracellular cAMP levels were performed using the cAMP Biotrak system (GE Life Sciences, Baie d’Urfe, QC).

### Insulin secretion assay

70% confluent INS832/13 cells were seeded in 24-well plates one day before use. On the day of the experiment, cells were washed and incubated for 30 min in 2.8 mM glucose KRBH buffer before incubation for 30 min at different glucose concentrations (2.8 mM and 16 mM) or 35 mM KCl to induce cell depolarization. At the end of the incubation, culture medium was collected, centrifuged to remove cells and assayed for insulin content by radioimmunoassay (Linco, St. Charles, MO). Results were normalized by mg of proteins.

Human GH secretion was determined as previously performed [24]. In brief, a plasmid encoding hGH is co-transfected with another plasmid of interest in a 1:3 ratio. hGH is known to co-localize with insulin and therefore its secretion matches that of insulin perfectly. With such a strategy, hormone release is measured exclusively in the transfected population of cells receiving the plasmid of interest and is not masked by secretion from the untransfected cells [24]. After 30 min of incubation as described above for insulin secretion, medium was collected for hGH ELISA and measured according to vendor protocol (Roche, Laval, QC).

### Glucose metabolism

Glucose oxidation was evaluated as ^14^CO_2_ production from [U-^14^C]-glucose. Briefly, INS cells were grown to confluence into 25 cm^2^ flasks and treated as described above under “insulin secretion assay” except that the incubation medium contained 25 µCi/ml of radio-labeled glucose tracer. Cellular metabolism was stopped by the addition of 1 ml of 40% perchloric acid after 30 min and ^14^CO_2_ was captured overnight by glass fiber filters previously soaked in 5% KOH.

### Intracellular calcium

Intracellular Ca^2+^ was measured in cells using Fura-2 (Life Technologies, Burlington, ON). Cells grown in 24-well plates were loaded with Fura-2, 30 min prior to treatment as described under “insulin secretion assay”. Results are displayed as 340 nm/380 nm ratios after background (auto-fluorescence) subtraction.

### Statistical analysis

Data are expressed as means ±SEM. Statistical analyses were performed using ANOVA or Student’s t test. We used a P<0.05 to declare statistically significant differences.

## Results

### FoxO1 regulates Ccn3 expression in pancreatic β-cells

We previously determined the transcriptional profile of pancreatic βINS832/13-cells overexpressing a constitutively active form of FoxO1 (CAFoxO1) using cDNA microarrays [10]. Our genomic analysis identified a transcript encoding *Ccn3* as one of the most significant genes whose expression was altered upon FoxO1 activation. We herein sought to identify and characterize *Ccn3* as a novel FoxO1 target and explore its biological role in β-cells.

**Figure 1:**
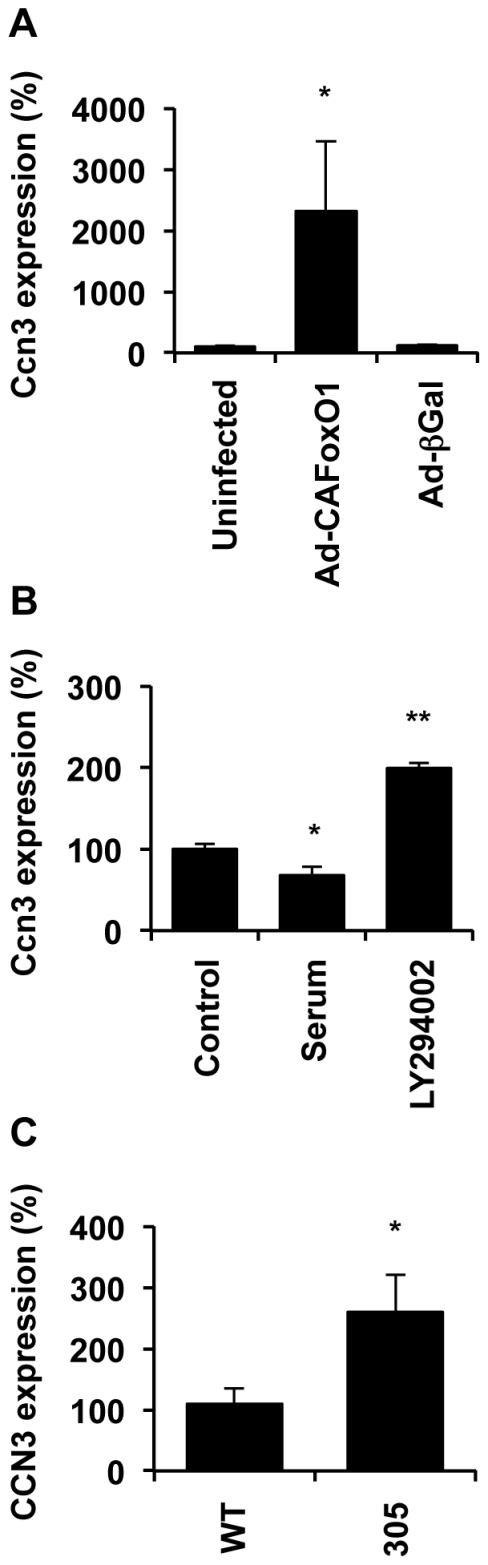
*Ccn3* is a transcriptional target of FoxO1 in β-cells. A) *Ccn3* expression by quantitative real-time qPCR in INS832/13 cells transduced with either Ad-β-Gal or Ad-CN-FoxO1 and cultured for 24 h. Results are means +/– SEM of 4 separate experiments. B) *Ccn3 *mRNA levels in INS832/13 cells treated with or without 10% serum and LY294002 (50 µM) for 4 h. Results represent means +/– SEM of 3 separate experiments. C) *Ccn3* expression in isolated islets from WT and transgenic mice with CAFoxO1 overexpression in their β-cells (called “305 mice”) (n = 5 for each). *, p<0.05.

Measurements of *Ccn3* expression by quantitative real-time PCR (qPCR) revealed CAFoxO1 significantly increased *Ccn3* mRNA levels compared to βGal after 24 h (Fig. 1A). Acute inhibition (4h) of FoxO1 by 10% serum decreased *Ccn3* mRNA levels by 40% compared to serum-deprived cells (Fig. 1B). Conversely, activation of FoxO1 via treatment with the PI3-kinase inhibitor LY294002 [8] increased *Ccn3* expression by 2-fold (Fig. 1B). These observations are consistent with a role of FoxO1 in *Ccn3* expression. We next sought to confirm our results in pancreatic islets isolated from WT or transgenic mice with FoxO1 gain-of-function. These mice, called “305 mice” and originally described in [6], express a FoxO1 protein mutated to include a single amino acid substitution replacing the phosphorylation site Ser253 with a non-phosphorylatable amino acid. Our data show that *Ccn3* expression was increased by approximately 3-fold in islets with constitutive FoxO1 activation (Fig. 1C).

**Figure 2:**
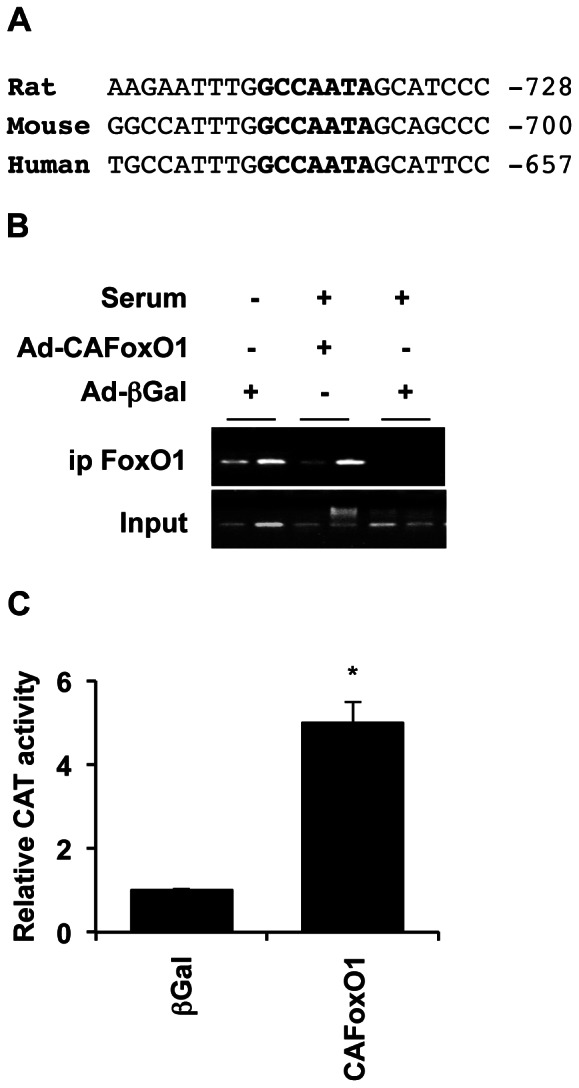
FoxO1 binds to a conserved element in the *Ccn3* promoter. A) A consensus Forkhead binding site (shown in bold) is conserved in the rat, mouse and human *Ccn3* promoters. Distance from the respective transcription start sites is indicated. B) FoxO1 was immunoprecipitated from cross-linked chromatin extracted from INS832/13 cells transduced with Ad-βGal or Ad-CAFoxO1 and cultured in the presence or absence of serum using an anti-FoxO1 antibody. Eluted DNA was PCR-amplified using oligonucleotides flanking the indicated forkhead site in the rat *Ccn3* promoter shown in (A). The figure shows duplicates for each condition. C) FoxO1 increased *Ccn3* promoter activity. INS cells were transiently transfected with a plasmid containing a *Ccn3* promoter-driven CAT construct concomitantly with either a plasmid encoding CAFoxO1 or control βGal. Lysates were prepared 24 h post-transfection for CAT assays. CAT activity was assigned an arbitrary value of 1 in βGal (control) samples. Results are means +/- SEM of 3 separate experiments. *, p<0.05.

A systematic search of the promoter of rat, mouse and human *Ccn3* orthologs revealed a putative conserved Forkhead response element (TATTGGC, defined in [25]) located ∼700 bp upstream of the transcription start site (Fig. 2A, reverse-complement sequence on the main DNA strand is shown). Chromatin immunoprecipitation experiments indicated that endogenous FoxO1 binds to a region of the *Ccn3* promoter encompassing this forkhead binding site, when activated following serum deprivation (Fig. 2B, two left-most bands). Endogenous FoxO1 could be displaced by the addition of serum, which is known to provoke FoxO1 nuclear exclusion [8] (two right-most bands), but not the constitutively active mutant, which bound DNA in the presence of serum (two middle bands). Consistently with our qPCR and ChIP assays, CAFoxO1 induced Ccn3 promoter activity in INS cells (Fig. 2C).

### Ccn3 expression is restricted to pancreatic islets and ducts, and increased in insulin resistance

**Figure 3:**
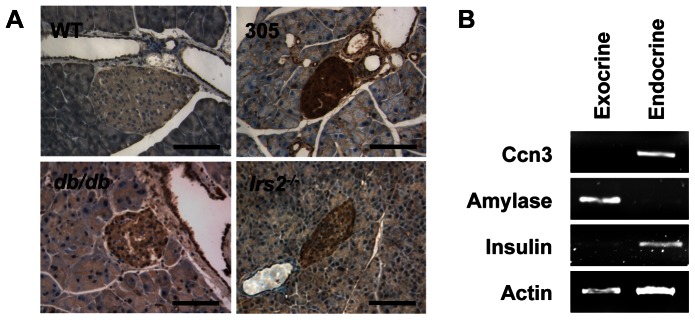
CCN3 is up-regulated in animal models of insulin resistance. A) We determined CCN3 protein levels in different genetic models of insulin resistance. We prepared paraffin sections from WT, CAFoxO1 transgenics (305), *db/db*, and *Irs2^–/–^* mice and performed CCN3 immunostaining (at least n = 3 for each). Representative images are shown. B) *Ccn3* expression in both the exocrine and the endocrine fraction was determined by PCR in 2 months old male animals. Amylase and insulin were used as specific exocrine and endocrine markers. Actin was used as a control in each sample. Representative images of 3 separate experiments are shown.

The precise tissue distribution of CCN3 in the pancreas has never been studied before. We therefore conducted immunohistochemistry on pancreas sections obtained from WT mice and genetic models of insulin resistance. Almost no detectable CCN3 expression could be observed in the pancreas of WT mice, indicating that CCN3 protein levels are low in control animals (Fig 3A, top left). However, CCN3 staining was increased in transgenic mice with CAFoxO1 overexpression (305 mice), consistent with the notion that *Ccn3* is upregulated by FoxO1 (Fig. 3A, top right). Although the increase in CCN3 staining could also reflect conformational changes that would render the epitope more accessible, we believe this is unlikely since we also detect the increase in Ccn3 at the mRNA levels in islets from 305 mice (Fig 1C). Interestingly, staining was found to be restricted to ducts and β-cells. We also observed increased CCN3 immunoreactivity in *db/db* and *Irs2^−/−^* mice, two genetic models of insulin resistance with β-cell failure. We next sought to confirm the tissue distribution of Ccn3 by PCR (Fig. 3B). *Ccn3* expression was undetectable in the exocrine tissue but present in the endocrine fraction of the pancreas. Amylase and insulin were used as controls.

### CCN3 is secreted from INS832/13 cells

**Figure 4:**
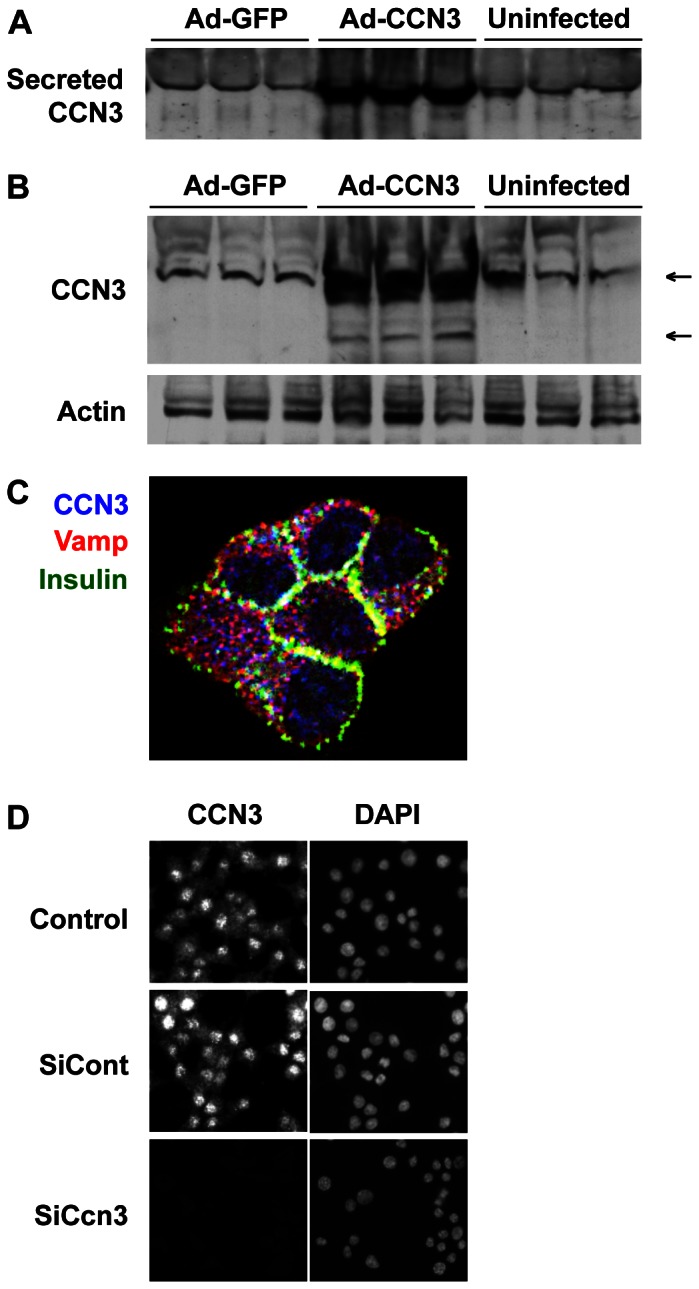
β INS832/13 cells secrete CCN3 protein. A–B) Western blot of CCN3 proteins in the media (A) or whole cell extracts (B) of either AdGFP or AdCCN3 infected cells and control uninfected cells. In (B) the top arrow indicate full-length CCN3 at a molecular weight of 47 KDa, whereas the bottom arrow indicate a CCN3 fragment of 35 KDa that have both been described in the literature. C) Immunohistochemical analysis of endogenous CCN3 protein localization in INS832/13 cells. We performed triple immunohistochemistry with CCN3 (blue), insulin (green) and Vamp/synaptobrevin (red) antibodies. D) Simple immunohistochemistry for CCN3 combined with DAPI staining demonstrate both nuclear and cytoplasmic localization of CCN3 in untransfected cells (Control, top) and cells transfected with a scrambled siRNAs (SiCont, middle). No CCN3 expression could be detected in cells transfected with Ccn3 siRNAs after 48 h (SiCcn3, bottom). Representative images of 3 separate experiments are shown.

Because CCN3 is an extra-cellular matrix protein, we sought to determine whether β-cells secrete CCN3 proteins. We thus transduced INS832/13 cells with adenovirus encoding either GFP or CCN3 and measured CCN3 protein levels in the medium by western blot. We detected endogenous CCN3 secretion in the medium of INS cells transduced with control AdGFP and the amount of CCN3 immunoreactivity was greater in the medium of INS cells transduced with AdCCN3 (Fig. 4A). This result suggests that β-cells endogenously express and secrete CCN3 proteins. Analysis of whole cell extracts by western blot revealed the presence of both full-length and cleaved products of CCN3 (Fig. 4B). The top and bottom arrows indicate the full length (47 KDa) and cleaved form (35 KDa) of CCN3, respectively. Cleaved CCN3 species were previously identified in various cell cultures but their biological functions have not been fully identified yet [14], [21]. We next analyzed CCN3 sub-cellular localization by immunofluorescence in INS832/13 cells (Fig. 4C). CCN3 co-localized with Vamp (Synaptobrevin), but not with insulin, indicating that CCN3 resides in a different subset of secretory granules than insulin. Accordingly, we failed to observe a regulation of CCN3 secretion by glucose (not shown) consistent with a constitutive CCN3 secretion via synaptic-like vesicles. In order to demonstrate the specificity of our anti-CCN3 antibodies, we have repeated our immunofluorescence staining in cells with siRNA-mediated *Ccn3* knockdown. Figure 4D shows that no CCN3 staining could be detected in siCcn3-transfected cells, compared to cells that were either transfected with scrambled siRNAs (siCont) or untransfected cells.

### CCN3 inhibits β-cell proliferation

**Figure 5:**
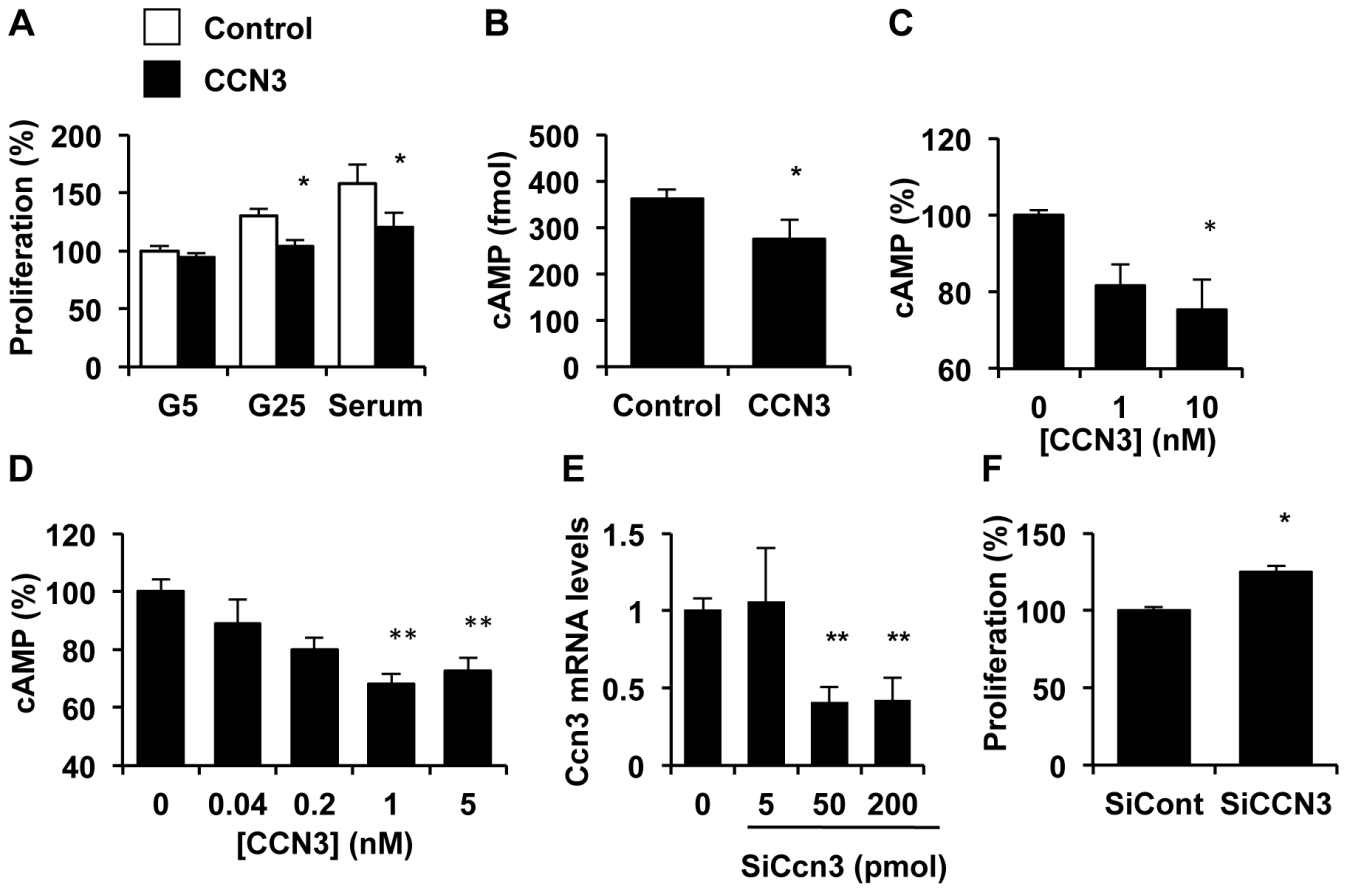
CCN3 protein decreases β-cell proliferation. A) The effect of CCN3 protein (1 nM) on β-cell proliferation was evaluated by BrdU incorporation in INS832/13 cells incubated at 5 mM glucose (G5), 25 mM glucose (G25) or in the presence of 10% serum. B) We studied the effects of 1 nM CCN3 protein on cAMP levels in INS cells by ELISA. C–D) Dose-dependent effects of CCN3 on cAMP levels in isolated rat islets (C) and INS cells (D). E) We measured *Ccn3* expression in cells transduced with increasing concentrations of siRNA specifically targeting *Ccn3* mRNA or scrambled siRNA as control by qPCR. F) Proliferation of INS832/13 cells transduced with either 50 pmol CCN3 or control siRNA. Results represent mean ± SEM of three separate experiments carried out at least in duplicate. *, p<0.05; **, p<0.01.

To investigate whether the increase in CCN3 levels are causally linked to impaired β-cell function in the etiology of diabetes/insulin resistance, we evaluated the effects of CCN3 on β INS832/13-cell proliferation. Treatment of cells with a physiological dose [26] of immunoaffinity-purified CCN3 proteins moderately but significantly reduced glucose- and serum-induced β-cell proliferation (Fig. 5A), as previously reported for other cell types [14], [27]. The inhibition of β-cell proliferation by CCN3 was associated with a reduction in cAMP levels (Fig. 5B) in INS832/13 cells. The effects of CCN3 on cAMP levels was confirmed in isolated rat islets (Fig. 5C). In INS832/13 cells, this effect was dose-dependent with a maximal effect observed at 1 nM (Fig. 5D). Conversely, silencing of *Ccn3* using specific siRNA increased β-cell proliferation by 20% as compared to cells treated with scrambled siRNA (Fig. 5E–F). Under our experimental conditions, *Ccn3* mRNA levels were decreased by 70% in cells transduced with specific CCN3 siRNA as compared to scrambled control siRNAs (Fig. 5E).

**Figure 6:**
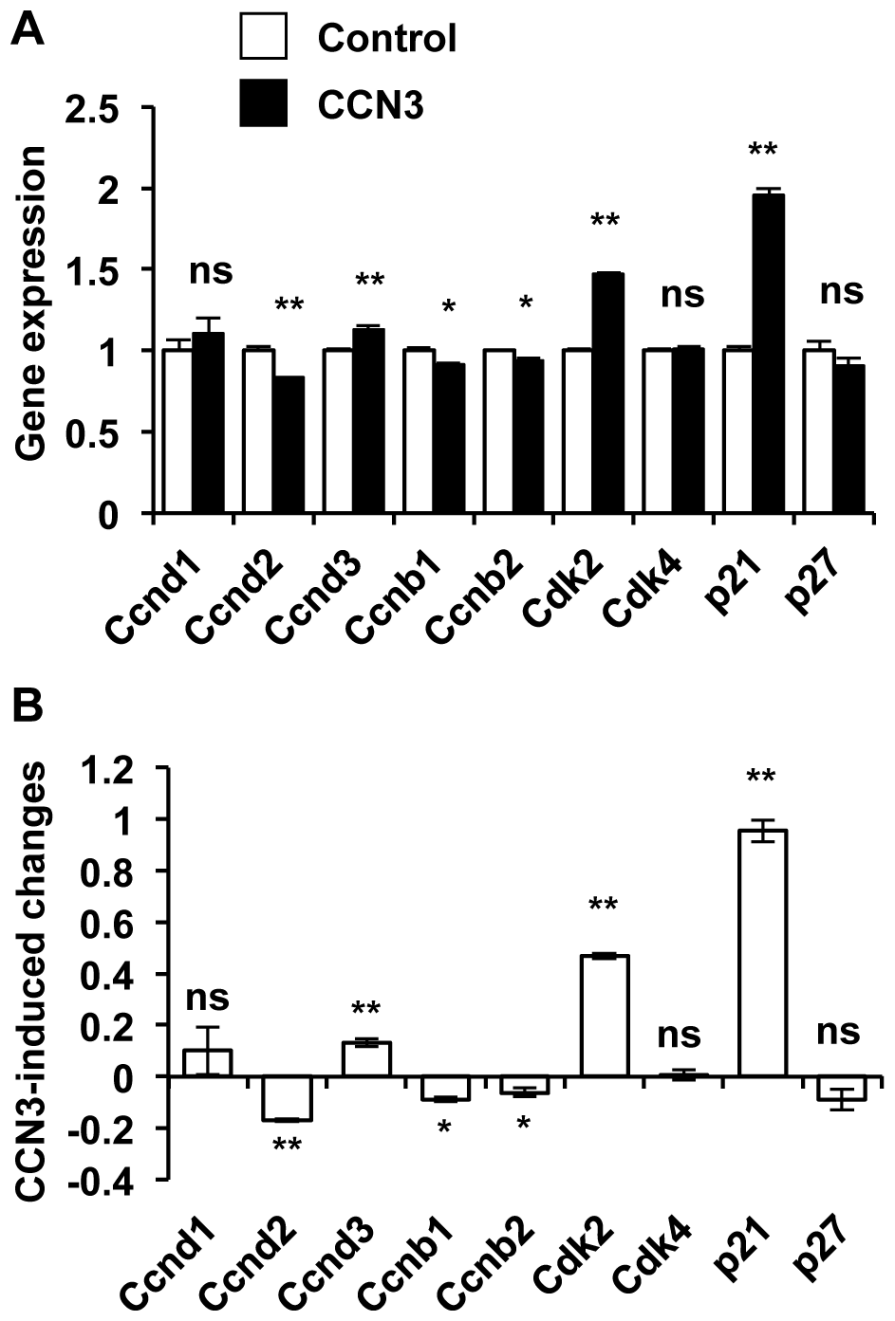
CCN3 modifies the expression of several genes involved in the control of cell cycle progression. A) mRNA levels of cells treated with or without CCN3 for 24 h. B) Reported changes in gene expression following CCN3 treatment. Results represent means +/– SEM of 3 separate experiments. *, p<0.05; **, p<0.01; ns  =  not significant.

In order to gain insight into the mechanism by which CCN3 could stunt β-cell proliferation, we have examined its effect on the expression levels of 84 key genes involved in cell cycle regulation using a commercial PCR array (from Qiagen). Our results revealed that CCN3 treatment alters the expression of several genes implicated in cell cycle progression. Figure 6 shows the expression levels of a selection of genes, which are known to be relevant for β-cell proliferation, in control cells and CCN-3 treated cells. It can be observed that CCN3 decreased the expression of *Ccnd2*, the most abundant cyclin D gene in β-cells, by 20%. *Ccnd3* was up-regulated by ∼15% but its expression level was roughly half of that of *Ccnd2*. CCN3 also slightly but significantly decreased the expression levels of both B-type cyclins, *Ccnb1* and *Ccnb2*. CCN3 treatment led to a 40% increase in Cdk2 expression but failed to affect Cdk4 expression, a Cdk protein that had been implicated in β-cell proliferation [28]. Importantly, CCN3 increased the expression of the cell cycle inhibitor p21 by 2-fold whereas p27 remained unchanged. Thus, the inhibitory effect of CCN3 on proliferation is consistent with its concerted actions on the expression of D-type as well as B-type cyclins, and p21.

### CCN3 impairs β-cell insulin secretion

**Figure 7:**
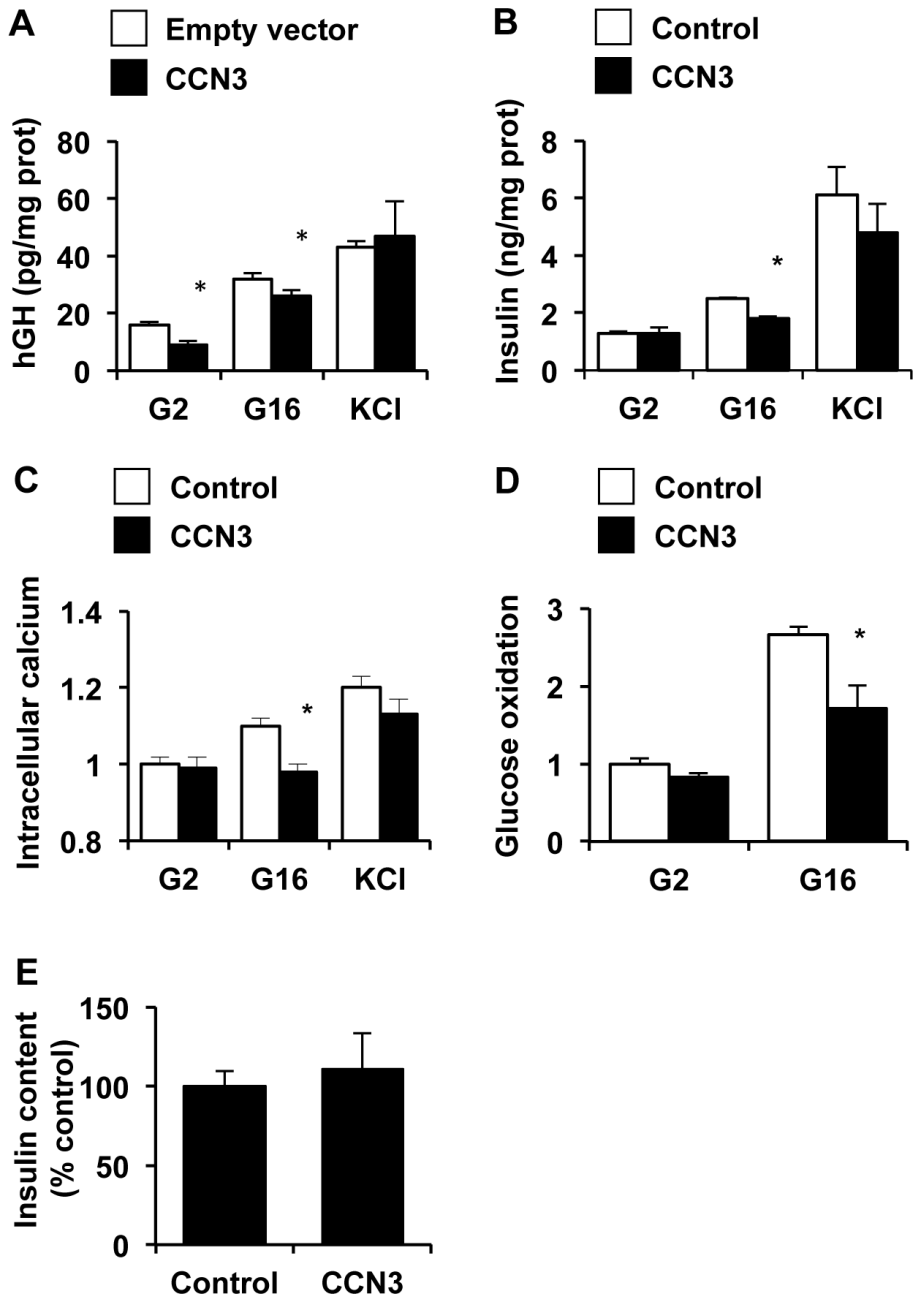
CCN3 impairs glucose-stimulated insulin secretion. A) We evaluated the effects of *Ccn3* overexpression on insulin secretion by the hGH co-transfection system. In brief, we measured the hGH levels in the media of INS832/13 cells co-transduced with hGH and CCN3 or the empty plasmid and incubated at 2.8 mM (G2.8) or 16 mM glucose (G16). KCl (35 mM) was used as control. B) We studied the effects of CCN3 protein on insulin secretion. Insulin released in the culture medium was measured following incubation of INS832/13 cells in 2.8 mM or 16 mM glucose/KRBH medium or in the presence of 35 mM KCl. Secreted insulin levels were normalized to protein content. C) Measurements of intracellular calcium in living INS832/13 cells treated as described in (B) using the Fura-2 dye. We measured the ratio of fluorescence signals produced by excitation of 340 nm and 380 nm and detected at 510 nm to determine intracellular calcium concentrations. D) Glucose oxidation was measured as ^14^CO_2_ production from [U-^14^C]-glucose in cells treated as described in (A) and the results were normalized to protein content. E) Cellular insulin content was measured by Elisa after lysis of the cells in acidic ethanol. Results represent mean ± SEM of three separate experiments carried out in triplicate. *, p<0.05.

We next studied the effects of CCN3 on insulin secretion. We examined glucose-induced insulin secretion in cells overexpressing *Ccn3* using the human growth hormone (hGH) cotransfection technique [24]. Because hGH secretion faithfully mirrors insulin secretion, cotransfection of *Ccn3* and the human growth hormone gene allows for evaluation of insulin secretion exclusively in transfected cells. *Ccn3* overexpression decreased glucose-induced hGH secretion (Fig. 7A). Similarly, treatment of cells with exogenous CCN3 proteins blunted glucose-induced insulin secretion without significantly affecting KCl-induced secretion (Fig. 7B). These observations suggests that CCN3 acts upstream of cell membrane depolarization and does not act through a non-specific effect on the exocytotic machinery. To confirm this hypothesis, we sought to examine the effects of CCN3 on intracellular calcium and glucose oxidation. Consistently, impairment of glucose-induced insulin secretion by CCN3 was accompanied by inhibition of both glucose-stimulated rise in intracellular calcium (Fig. 7C) and glucose oxidation (Fig. 7D), without affecting insulin content (Fig. 7E).

## Discussion

FoxO1 is a prominent mediator of insulin/IGF signaling in pancreatic β-cells. FoxO1 controls a variety of cellular processes including cell proliferation, differentiation, metabolism and stress resistance [5]. We have previously performed a genomic analysis of FoxO1 targets in β-cells in order to investigate the mechanisms underlying β-cell adaptation to insulin resistance. In the present study, we identify for the first time *Nephroblastoma overexpressed gene (Nov/Ccn3)* as a novel transcriptional target of FoxO1 in β-cells. Promoter analysis and chromatin immunoprecipitation demonstrate that FoxO1 binds to an evolutionarily conserved element in the *Ccn3* promoter to stimulate its expression. Accordingly, we show that expression of *Ccn3* is increased in genetic mouse models of insulin resistance. Moreover, Ccn3 expression is increased in lymphocytes from patients with obesity. We also explored for the first time the role of CCN3 proteins in β-cell function. Our biochemical studies provide evidence that CCN3 decreases β-cell proliferation and stunts glucose oxidation, which translates into reduced glucose-induced insulin secretion. The role of CCN3 in β-cell mass and function as well as in insulin resistance could be of great clinical importance.

*CCN3* is expressed in a selection of adult tissues, including prostate, testes, heart, brain, peripheral lymphocytes and pancreas [29]. However, the exact tissue distribution of CCN3 in the pancreas had never been investigated. Here, we show for the first time that in the adult mouse pancreas, *CCN3* expression is restricted to ducts and β-cells. The previous detection of CCN3 in pancreatic ducts during embryonic development [30] raises the possibility that CCN3 could play a role in duct and/or islet cell differentiation. CCN3 has been previously shown to inhibit myoblast [31], [32] as well as osteoblast differentiation [33]. Also, CCN3 is essential for primitive hematopoietic progenitor cell activity [34]. Thus, there is a precedent for an effect of CCN3 in the control of cell differentiation.

The circulating levels of CCN3 were previously estimated to ∼7 nM in adults with a significant increase with age [26]. Interestingly, ageing is a well-known risk factor for the development of insulin resistance. Whether circulating CCN3 levels are higher in diabetic and insulin resistant patients remains to be tested. A previous genomic study comparing the expression profile of islets from healthy donors to patients with type 2 diabetes reported a ∼2-fold increase in *Ccn3* mRNA levels in diabetes ([35] and G.C. Weir, personal communication). To date, the origin of the circulating CCN3 proteins remains elusive. In this study, we show that β-cells produce the secreted (full-length) form of CCN3 and β INS cells endogenously secrete CCN3 proteins in the medium. These results raise the possibility that insulin resistant β-cells could represent an important origin of circulating CCN3.

The fact that CCN3 is up-regulated in insulin resistance and secreted by pancreatic β-cells is consistent with a model in which circulating CCN3 would act locally as well as distally to mediate the adaptation to this metabolic stress. In β-cells CCN3 blunts proliferation and insulin secretion, presumably as a protective mechanism to prevent exhaustion [36], [37]. However, it is possible that prolonged inhibition of β-cell mass and function by CCN3 in the face of insulin resistance would lead to β-cell failure, thus linking insulin resistance to progressive deterioration of β-cell function. The metabolic effects of CCN3 in peripheral tissues have never been investigated. These important studies would be facilitated by the identification of a CCN3 receptor. However, the CCN3 receptor (if any) has yet to be identified [12].

There is a growing interest into the potential roles of CCN family members in β-cell function [38]. Undoubtedly, CCN2/CTGF constitutes the most widely studied CCN protein in β-cells in health and disease. Immunohistochemical analysis of CCN2 expression in mouse embryonic pancreata suggested a role in β-cell development [39]. Indeed, in the mouse embryo, CCN2 is expressed in pancreatic ductal and vascular endothelium, as well as in developing insulin-positive cells. Importantly, CCN2 expression was found to coincide with islet morphogenesis and to terminate soon after birth. Moreover, CCN2 null embryos displayed reduced β-cell mass, which was attributed to decreased proliferation. Conversely, CCN2 overexpression in β-cells during embryogenesis, using a doxycycline-inducible system in which the rtTA was driven by the rat insulin promoter, increased islet mass at birth by promoting proliferation of developing β-cells [40]. Collectively, these studies along with those suggesting a role for CCN proteins in inflammatory responses [41] highlight their potential as therapeutic targets in diabetes.

In summary, our results identify *Ccn3* as a novel transcriptional target of FoxO1 that could underlie the adaptive response to insulin resistance. Further studies with genetically-engineered mice with either loss- or gain-of-function mutations would be required to further explore the role of CCN3 in insulin resistance and β-cell function. Such studies might validate CCN3 as a pharmacological target in the treatment of diabetes. Certainly, our results warrant closer examination of the role of CCN3 in diabetes.

## References

[1] Drucker DJ (2006) The biology of incretin hormones. Cell Metab 3: 153–165. 16517403 10.1016/j.cmet.2006.01.004

[2] Weir GC, Laybutt DR, Kaneto H, Bonner-Weir S, Sharma A (2001) Beta-cell adaptation and decompensation during the progression of diabetes. Diabetes 50 Suppl 1S154–159. 11272180 10.2337/diabetes.50.2007.s154

[3] Porte D Jr, Kahn SE (2001) beta-cell dysfunction and failure in type 2 diabetes: potential mechanisms. Diabetes 50 Suppl 1S160–163. 11272181 10.2337/diabetes.50.2007.s160

[4] Hribal ML, Oriente F, Accili D (2002) Mouse models of insulin resistance. Am J Physiol Endocrinol Metab 282: E977–981. 11934661 10.1152/ajpendo.00561.2001

[5] Accili D, Arden KC (2004) FoxOs at the crossroads of cellular metabolism, differentiation, and transformation. Cell 117: 421–426. 15137936 10.1016/s0092-8674(04)00452-0

[6] Nakae J, Biggs WH, 3rd, Kitamura T, Cavenee WK, Wright CV, et al (2002) Regulation of insulin action and pancreatic beta-cell function by mutated alleles of the gene encoding forkhead transcription factor Foxo1. Nat Genet 32: 245–253. 12219087 10.1038/ng890

[7] Kitamura T, Nakae J, Kitamura Y, Kido Y, Biggs WH, 3rd, et al (2002) The forkhead transcription factor Foxo1 links insulin signaling to Pdx1 regulation of pancreatic beta cell growth. J Clin Invest 110: 1839–1847. 12488434 10.1172/JCI200216857 PMC151657

[8] Buteau J, Spatz ML, Accili D (2006) Transcription factor FoxO1 mediates glucagon-like peptide-1 effects on pancreatic beta-cell mass. Diabetes 55: 1190–1196. 16644672 10.2337/db05-0825

[9] Kitamura YI, Kitamura T, Kruse JP, Raum JC, Stein R, et al (2005) FoxO1 protects against pancreatic beta cell failure through NeuroD and MafA induction. Cell Metab 2: 153–163. 16154098 10.1016/j.cmet.2005.08.004

[10] Buteau J, Shlien A, Foisy S, Accili D (2007) Metabolic diapause in pancreatic beta-cells expressing a gain-of-function mutant of the forkhead protein Foxo1. J Biol Chem 282: 287–293. 17107961 10.1074/jbc.M606118200

[11] Buteau J, Accili D (2007) Regulation of pancreatic beta-cell function by the forkhead protein FoxO1. Diabetes Obes Metab 9 Suppl 2140–146. 17919188 10.1111/j.1463-1326.2007.00782.x

[12] Perbal B (2006) NOV story: the way to CCN3. Cell Commun Signal 4: 3. 16504021 10.1186/1478-811X-4-3 PMC1434737

[13] Perbal B (2001) NOV (nephroblastoma overexpressed) and the CCN family of genes: structural and functional issues. Mol Pathol 54: 57–79. 11322167 10.1136/mp.54.2.57 PMC1187006

[14] Holbourn KP, Acharya KR, Perbal B (2008) The CCN family of proteins: structure-function relationships. Trends Biochem Sci 33: 461–473. 18789696 10.1016/j.tibs.2008.07.006 PMC2683937

[15] Perbal B (2004) CCN proteins: multifunctional signalling regulators. Lancet 363: 62–64. 14723997 10.1016/S0140-6736(03)15172-0

[16] Wiltshire S, Hattersley AT, Hitman GA, Walker M, Levy JC, et al (2001) A genomewide scan for loci predisposing to type 2 diabetes in a U.K. population (the Diabetes UK Warren 2 Repository): analysis of 573 pedigrees provides independent replication of a susceptibility locus on chromosome 1q. Am J Hum Genet 69: 553–569. 11484155 10.1086/323249 PMC1235485

[17] Vaessen N, Heutink P, Houwing-Duistermaat JJ, Snijders PJ, Rademaker T, et al (2002) A genome-wide search for linkage-disequilibrium with type 1 diabetes in a recent genetically isolated population from the Netherlands. Diabetes 51: 856–859. 11872691 10.2337/diabetes.51.3.856

[18] Sale MM, FitzGerald LM, Charlesworth JC, Bowden DW, Rich SS (2002) Evidence for a novel type 1 diabetes susceptibility locus on chromosome 8. Diabetes 51 Suppl 3S316–319. 12475769 10.2337/diabetes.51.2007.s316

[19] Frayling TM, Wiltshire S, Hitman GA, Walker M, Levy JC, et al (2003) Young-onset type 2 diabetes families are the major contributors to genetic loci in the Diabetes UK Warren 2 genome scan and identify putative novel loci on chromosomes 8q21, 21q22, and 22q11. Diabetes 52: 1857–1863. 12829657 10.2337/diabetes.52.7.1857

[20] An P, Freedman BI, Rich SS, Mandel SA, Arnett DK, et al (2006) Quantitative trait loci on chromosome 8q24 for pancreatic beta-cell function and 7q11 for insulin sensitivity in obese nondiabetic white and black families: evidence from genome-wide linkage scans in the NHLBI Hypertension Genetic Epidemiology Network (HyperGEN) study. Diabetes 55: 551–558. 16443794 10.2337/diabetes.55.02.06.db05-0714

[21] Kyurkchiev S, Yeger H, Bleau AM, Perbal B (2004) Potential cellular conformations of the CCN3(NOV) protein. Cell Commun Signal 2: 9. 15361251 10.1186/1478-811X-2-9 PMC519031

[22] Hohmeier HE, Mulder H, Chen G, Henkel-Rieger R, Prentki M, et al (2000) Isolation of INS-1-derived cell lines with robust ATP-sensitive K+ channel-dependent and -independent glucose-stimulated insulin secretion. Diabetes 49: 424–430. 10868964 10.2337/diabetes.49.3.424

[23] Martinerie C, Chevalier G, Rauscher FJ, 3rd, Perbal B (1996) Regulation of nov by WT1: a potential role for nov in nephrogenesis. Oncogene 12: 1479–1492. 8622864

[24] Antinozzi PA, Garcia-Diaz A, Hu C, Rothman JE (2006) Functional mapping of disease susceptibility loci using cell biology. Proc Natl Acad Sci U S A 103: 3698–3703. 16537450 10.1073/pnas.0510521103 PMC1533777

[25] Ramaswamy S, Nakamura N, Sansal I, Bergeron L, Sellers WR (2002) A novel mechanism of gene regulation and tumor suppression by the transcription factor FKHR. Cancer Cell 2: 81–91. 12150827 10.1016/s1535-6108(02)00086-7

[26] Thibout H, Martinerie C, Creminon C, Godeau F, Boudou P, et al (2003) Characterization of human NOV in biological fluids: an enzyme immunoassay for the quantification of human NOV in sera from patients with diseases of the adrenal gland and of the nervous system. J Clin Endocrinol Metab 88: 327–336. 12519873 10.1210/jc.2002-020304

[27] Bleau AM, Planque N, Lazar N, Zambelli D, Ori A, et al (2007) Antiproliferative activity of CCN3: involvement of the C-terminal module and post-translational regulation. J Cell Biochem 101: 1475–1491. 17340618 10.1002/jcb.21262

[28] Rane SG, Dubus P, Mettus RV, Galbreath EJ, Boden G, et al (1999) Loss of Cdk4 expression causes insulin-deficient diabetes and Cdk4 activation results in beta-islet cell hyperplasia. Nat Genet 22: 44–52. 10319860 10.1038/8751

[29] Burren CP, Wilson EM, Hwa V, Oh Y, Rosenfeld RG (1999) Binding properties and distribution of insulin-like growth factor binding protein-related protein 3 (IGFBP-rP3/NovH), an additional member of the IGFBP Superfamily. J Clin Endocrinol Metab 84: 1096–1103. 10084601 10.1210/jcem.84.3.5577

[30] Kocialkowski S, Yeger H, Kingdom J, Perbal B, Schofield PN (2001) Expression of the human NOV gene in first trimester fetal tissues. Anat Embryol (Berl) 203: 417–427. 11453160 10.1007/s004290100177

[31] Calhabeu F, Lafont J, Le Dreau G, Laurent M, Kazazian C, et al (2006) NOV/CCN3 impairs muscle cell commitment and differentiation. Exp Cell Res 312: 1876–1889. 16600215 10.1016/j.yexcr.2006.02.027

[32] Sakamoto K, Yamaguchi S, Ando R, Miyawaki A, Kabasawa Y, et al (2002) The nephroblastoma overexpressed gene (NOV/ccn3) protein associates with Notch1 extracellular domain and inhibits myoblast differentiation via Notch signaling pathway. J Biol Chem 277: 29399–29405. 12050162 10.1074/jbc.M203727200

[33] Minamizato T, Sakamoto K, Liu T, Kokubo H, Katsube K, et al (2007) CCN3/NOV inhibits BMP-2-induced osteoblast differentiation by interacting with BMP and Notch signaling pathways. Biochem Biophys Res Commun 354: 567–573. 17250806 10.1016/j.bbrc.2007.01.029

[34] Gupta R, Hong D, Iborra F, Sarno S, Enver T (2007) NOV (CCN3) functions as a regulator of human hematopoietic stem or progenitor cells. Science 316: 590–593. 17463287 10.1126/science.1136031

[35] Marselli L, Thorne J, Dahiya S, Sgroi DC, Sharma A, et al (2010) Gene expression profiles of Beta-cell enriched tissue obtained by laser capture microdissection from subjects with type 2 diabetes. PLoS One 5: e11499. 20644627 10.1371/journal.pone.0011499 PMC2903480

[36] Ritzel RA, Hansen JB, Veldhuis JD, Butler PC (2004) Induction of beta-cell rest by a Kir6.2/SUR1-selective K(ATP)-channel opener preserves beta-cell insulin stores and insulin secretion in human islets cultured at high (11 mM) glucose. J Clin Endocrinol Metab 89: 795–805. 14764798 10.1210/jc.2003-031120

[37] Ritzel RA, Butler PC (2003) Replication increases beta-cell vulnerability to human islet amyloid polypeptide-induced apoptosis. Diabetes 52: 1701–1708. 12829636 10.2337/diabetes.52.7.1701

[38] Charrier A, Brigstock DR (2013) Regulation of pancreatic function by connective tissue growth factor (CTGF, CCN2). Cytokine Growth Factor Rev 24: 59–68. 22884427 10.1016/j.cytogfr.2012.07.001 PMC3508350

[39] Crawford LA, Guney MA, Oh YA, Deyoung RA, Valenzuela DM, et al (2009) Connective tissue growth factor (CTGF) inactivation leads to defects in islet cell lineage allocation and beta-cell proliferation during embryogenesis. Mol Endocrinol 23: 324–336. 19131512 10.1210/me.2008-0045 PMC2654514

[40] Guney MA, Petersen CP, Boustani A, Duncan MR, Gunasekaran U, et al (2011) Connective tissue growth factor acts within both endothelial cells and beta cells to promote proliferation of developing beta cells. Proc Natl Acad Sci U S A 108: 15242–15247. 21876171 10.1073/pnas.1100072108 PMC3174622

[41] Jun JI, Lau LF (2011) Taking aim at the extracellular matrix: CCN proteins as emerging therapeutic targets. Nat Rev Drug Discov 10: 945–963. 22129992 10.1038/nrd3599 PMC3663145

